# Pembrolizumab for the treatment of progressive multifocal leukoencephalopathy following anti‐CD19 CAR‐T therapy: a case report

**DOI:** 10.1002/jha2.274

**Published:** 2021-08-04

**Authors:** Strachan Mackenzie, Manar Shafat, Harriet Roddy, Harpreet Hyare, Lorna Neill, Maria A. V. Marzolini, Michael Gilhooley, Teresa Marafioti, Eleanna Kara, Emilie Sanchez, Jeremy Rees, David S. Lynch, Kirsty Thomson, Kirit M. Ardeshna, Arian Laurence, Karl S. Peggs, Maeve O'Reilly, Claire Roddie

**Affiliations:** ^1^ UCL Cancer Institute Paul O'Gorman Building University College London London UK; ^2^ UCL Great Ormond Street Institute of Child Health University College London London UK; ^3^ Department of Haematology University College London Hospital NHS Foundation Trust London UK; ^4^ The Institute of Ophthalmology University College London Bath Street London UK; ^5^ Department of Histopathology University College London 60 Whitfield Street London UK; ^6^ Department of Neuropathology National Hospital of Neurology and Neurosurgery University College London Hospital NHS Foundation Trust Queen Square London UK; ^7^ Department of Virology University College London Hospital NHS Foundation Trust London UK; ^8^ Institute of Neurology National Hospital of Neurology and Neurosurgery University College London Queen Square London UK; ^9^ Department of Radiology University College London Hospital NHS Foundation Trust London UK

**Keywords:** checkpoint inhibition, chimeric antigen receptor T‐ cell, herald lesion, immune reconstitution inflammatory syndrome, pembrolizumab, progressive multifocal leukoencephalopathy

## Abstract

Progressive multifocal leukoencephalopathy (PML) is an opportunistic brain infection with few treatment options and poor survival when reversal of the underlying immune dysfunction is not achievable. JC polyomavirus reactivation resulting in PML can rarely complicate chimeric antigen receptor T‐cell (CAR‐T) therapy. We describe successful treatment of PML with Programmed death‐1 (PD‐1) blockade using pembrolizumab, 4 months following axicabtagene ciloleucel. Radiological features of immune reconstitution inflammatory syndrome without clinical deterioration were seen. Evidence of anti‐viral immune reconstitution by in vitro detection of JC‐specific T‐cells and sustained neurological recovery in this patient suggest PD‐1 blockade may be an effective treatment approach for PML post‐CAR‐T.

## 1 INTRODUCTION

CD19‐targeted chimeric antigen receptor T‐cell (CAR‐T) therapy has revolutionised the treatment of aggressive B‐cell malignancies but is associated with unique toxicities including cytokine release syndrome and immune effector cell‐mediated neurological syndrome (ICANS). B‐cell aplasia and resultant hypogammaglobulinaemia are also common and confer an increased risk of viral infection [1].

Progressive multifocal leukoencephalopathy (PML) is a severe, demyelinating infection of the central nervous system caused by the reactivation of latent JC polyomavirus (JCV). Profound immunosuppression allows replication‐driven neurotropic JCV variants to cause lytic infection of oligodendrocytes/glial cells, leading to progressive neurological decline and short survival unless immune reconstitution occurs [2]. Of the anti‐viral pharmacotherapies that have been prospectively trialled (cytarabine, cidofovir, mefloquine), none have shown clinical benefit [2]. Mirtazapine, a 5‐HT2A receptor antagonist, can block JCV entry into glial cells and although no survival benefit has been demonstrated, it has been recommended at diagnosis [3]. Adoptive BK virus‐specific cytotoxic T‐lymphocytes (CTL) have been used successfully in three cases [4]. Expression of the inhibitory Programmed death‐1 (PD‐1) co‐receptor is high on T‐cells isolated from patients with PML [5], and the use of PD‐1 checkpoint inhibitors to reinvigorate JCV‐specific T‐cell responses has shown promise [6, 7, 8, 9].

Here we describe the first reported case of PML post‐CAR‐T therapy treated successfully with PD‐1 blockade.

## 2 CASE DESCRIPTION

**FIGURE 1:**
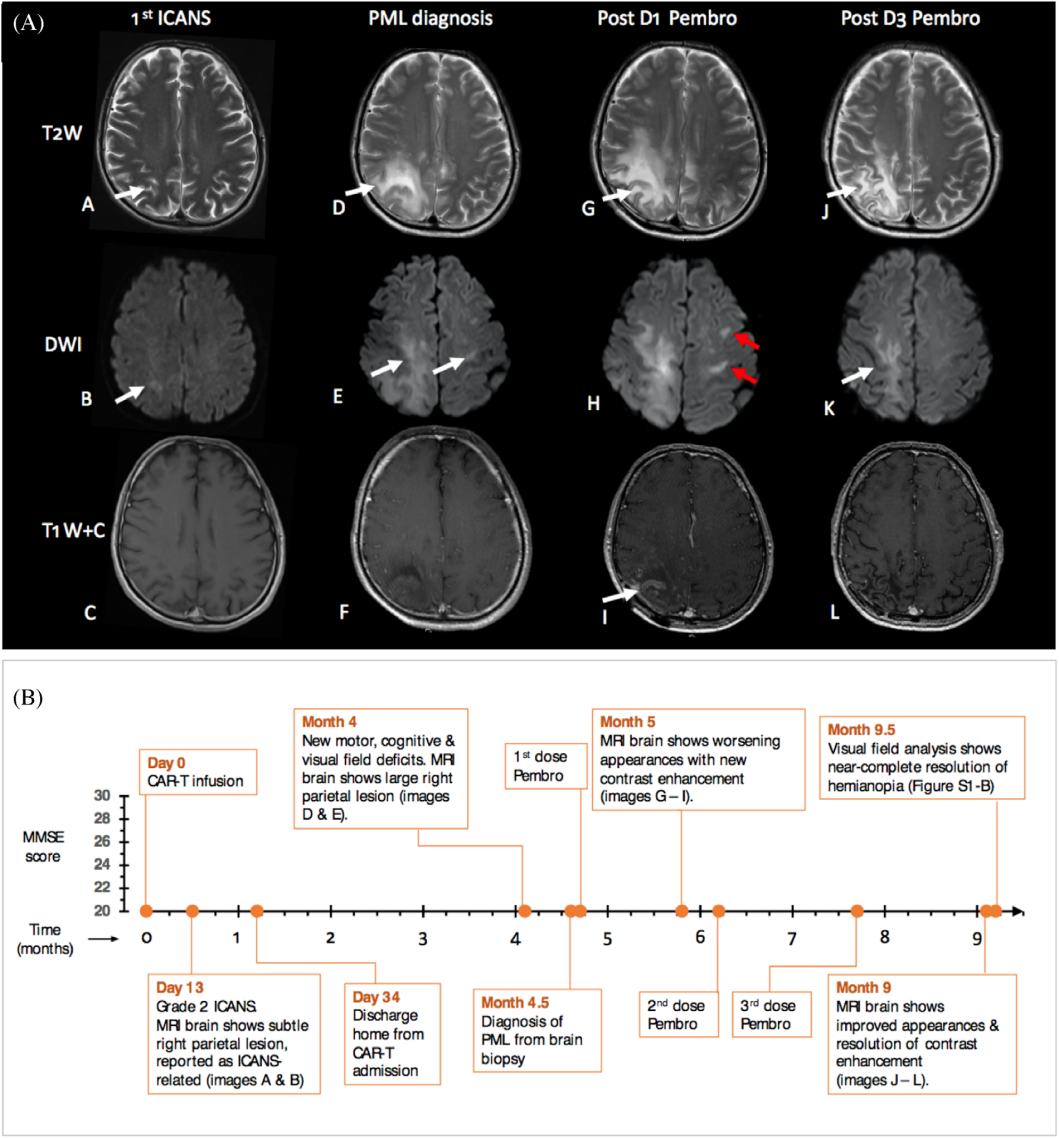
**(A) Magnetic resonance imaging of the brain**. Axial, gadolinium‐enhanced images showing T2‐weighted (T2W), diffusion‐weighted (DWI) and contrast‐enhanced (T1W+C) sections at 1st episode of immune effector cell‐mediated neurological syndrome (ICANS; A–C), progressive multifocal leukoencephalopathy (PML) diagnosis (D–F), 1 month following 1st pembrolizumab dose (D1; G–I), and after 3rd dose (D3) of pembrolizumab (J–L), which was 5 months after 1st infusion. Panels A and B show a small focus of T2‐weighted signal abnormality in the right deep parietal white matter, demonstrating mild restriction of diffusion on DWI (B) at ICANS diagnosis. After the first pembrolizumab dose, note the relative increase in the size of PML lesions (G) with the progression of restricted diffusion (H) including new contralateral areas (red arrows) and new contrast enhancement (I), which had improved (J, K) or resolved (L) on interval imaging a month after D3. **(B) Timeline of clinical and radiological events post CAR‐T infusion (x‐axis) with serial mini‐mental state examination (MMSE) scores (y‐axis)**. At presentation, MMSE score was reduced (25/30), declined further after 2 weeks (22/30), but improved over 4 months (to 29/30) following pembrolizumab infusion. Image references refer to (A)

A 41‐year‐old man with bulky mediastinal large B‐cell lymphoma (LBCL), refractory to R‐CHOP, ICE, and R‐DHAP chemotherapy was referred for CD19‐CAR‐T therapy. Bridging comprised gemcitabine/cisplatin and axicabtagene ciloleucel (axi‐cel) was infused following lymphodepletion with fludarabine and cyclophosphamide. Excellent CAR‐T expansion was observed (Figure S3). Complications included Grade2 cytokine release syndrome, treated with tocilizumab, and two episodes of Grade 2 ICANS on days 13 and 26 (expressive dysphasia, confusion, and immune effector cell‐associated encephalopathy (ICE) score 6/10), which resolved rapidly with corticosteroids. MRI imaging (day 13) showed a small T2‐weighted hyperintense lesion in the right parietal white matter (WM), reported as cytotoxic oedema secondary to ICANS (Figure 1A, A–B). A PET‐CT at month 3 post‐infusion confirmed complete metabolic remission (CMR).

Four months following axi‐cel infusion, he reported one week of headache, confusion, visuospatial difficulties, and left arm clumsiness. Neurological examination identified left‐sided dyspraxia, pronator drift, and homonymous hemianopia (Figure S1) with neglect and optic ataxia. Mini‐mental state examination (MMSE) score was 25/30 (Figure 1B), with constructional apraxia (Figure S2).

MRI brain showed multifocal WM lesions with a prominent right parietal lesion at the site of the prior ‘original’ WM lesion (Figure 1A, D–F). Extensive cerebrospinal fluid (CSF) investigations including JCV PCR were negative (lower limit of detection: 50 copies/ml). Bloodwork revealed ongoing CAR‐T persistence (Figure S3), cytopenias (neutrophils, 1 × 10^9^/L [filgrastim‐dependent], CD4+, 170 cells/μl), and hypogammaglobulinaemia (IgG, 1.9 g/L).

**FIGURE 2:**
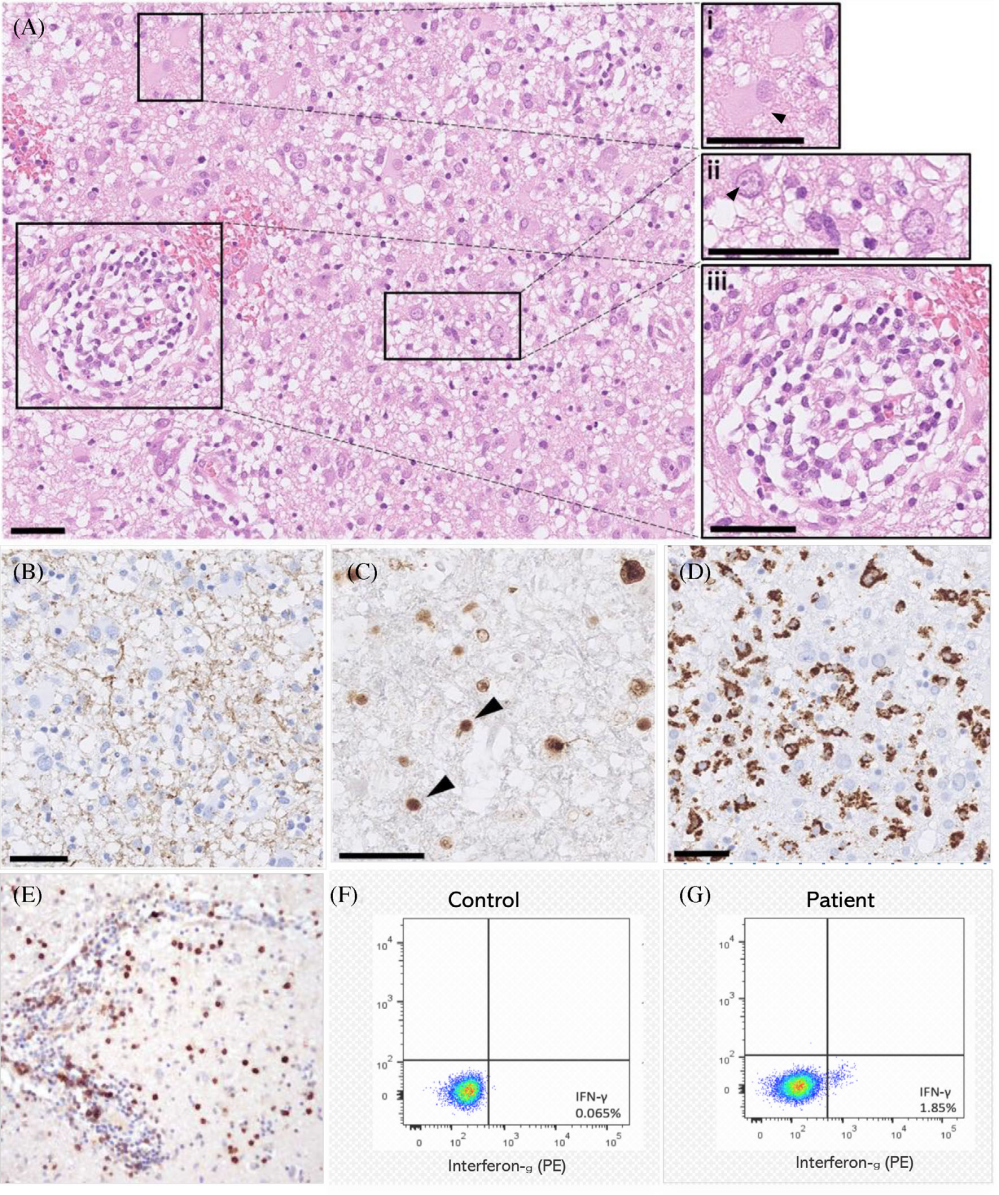
**(A–E)** **Histopathology of brain biopsy from the right parietal lobe**. Subcortical white matter demonstrated the classic PML diagnostic triad of 1. ‘bizarre’ astrocytes ((A), inset i arrowhead, hematoxylin & eosin (H&E) stain) 2. enlarged oligodendrocyte nuclei (inset ii) which contained basophilic viral inclusions (arrowhead), and 3. demyelination ((B), SMI94 immunohistochemistry). The intense macrophage infiltrate ((D) CD68 immunohistochemistry) with relatively preserved axons (not shown) were suggestive of inflammatory demyelination. SV40 immunohistochemistry for JC virus (C) showed numerous intranuclear inclusions (arrowheads) also consistent with PML. Perivascular inflammation was visible on the H&E‐stained sections ((A), inset iii) and PD‐1‐positive cells were present in the intravascular and perivascular spaces ((E), PD‐1 immunohistochemistry), and throughout the parenchyma (not shown). PD‐1 positive cells made up 20% of total nucleated cells. Scale bars = 50 μm. **(F, G)** **In vitro** **detection of JCV‐specific T cells**. After the 3rd dose of pembrolizumab, following exposure to JCV antigen peptides, intracellular IFN‐γ secretion was detected in a small population of T‐cells by flow cytometry (1.85% [of live CD3+ cells] in the patient (G) compared to <0.1% in a healthy control (F)), suggesting a cellular anti‐JCV immune response post‐pembrolizumab. Briefly, peripheral blood mononuclear cells were pulsed with a JCV peptide pool of VP1, VP2, VP3, ST and LT (Miltenyi Biotec) each at a concentration of 1μg/ml, cultured in media supplemented with 5% human AB serum, and IL‐7 & IL‐15 at 10 ng/ml, over 7 days

He deteriorated further over 5 days, developing a focal seizure with secondary generalisation, left hemiparesis (power 4/5), and a further reduction in MMSE (22/30). Diagnostic brain biopsy demonstrated JCV‐mediated PML with inflammatory demyelination and 20% PD‐1 positivity (Figure 2A–E). We commenced mirtazapine and three doses of pembrolizumab 200 mg (2.4 mg/kg) intravenously every 4–6 weeks in line with Cortese et al. [8]

Following dose 1, the MRI brain revealed worsening appearances, with new foci of contrast enhancement and restricted diffusion (Figure 1A, G–I), but the neurological examination was unchanged, suggestive of ‘radiological’ PML‐immune reconstitution inflammatory syndrome (PML‐IRIS) [2, 10]. We, therefore, continued pembrolizumab.

Over 5 months following dose 1, we observed neurological recovery, with a near‐complete resolution of weakness, dyspraxia (Figure S2), and hemianopia (Figure S1), and improved MMSE (29/30, Figure 1B) and neuroimaging appearances (Figure 1A, J–L). CD4+ count recovered (560 cells/μl). We detected JCV‐specific T‐cells in the peripheral blood (Figure 2G), which has been associated with response to PD‐1 blockade in PML [8]. Eighteen months post PML diagnosis, the patient remains neurologically stable, has returned to normal activity, and remains in CMR.

## 3 DISCUSSION

To date, two other published cases also report PML following CAR‐T for LBCL, but we report the first successful use of pembrolizumab.

The first case describes a 68‐year‐old woman, diagnosed with PML 7 months post‐axi‐cel, in whom clinical and radiological stabilization of PML (but not clearance of blood/CSF viremia) was achieved with mirtazapine, mefloquine, IVIG, and ultimately CTL immunotherapy [11]. The second case describes a 61‐year‐old woman diagnosed with PML 14 months post‐axi‐cel, but who died from sepsis prior to initiation of therapy [12]. Common features with our case include LBCL treated with axi‐cel to CMR, exposure to multiple prior lines of therapy (median 4, range 3–4; prior autologous stem cell transplantation (SCT) in Cases 1 and 2), persistent CD4+ lymphopenia (<200 cells/μl), and hypogammaglobulinaemia (<4 g/L).

PML arising post‐CAR‐T is likely to be a cumulative effect of multiple immunosuppressive insults, including lymphodepletion with fludarabine [13], corticosteroids for CAR‐T immunotoxicity [14], post‐CAR CD4+ lymphopenia [2], and B‐cell aplasia/hypogammaglobulinaemia. Further, patients with LBCL are at higher risk of PML due to their underlying lymphoproliferative disease, prior SCT, and rituximab exposure [2]. PML arising post‐CAR‐T is therefore likely a combined risk, determined by pre‐infusion and CAR‐T‐related factors.

Our case highlighted several learning points. First, negative JCV PCR in CSF should not preclude further investigation of PML using brain biopsy if there is clinical suspicion. Second, the unilateral lesion on MRI at day 13, in retrospect, was potentially a PML ‘herald’ lesion rather than an ICANS‐associated WM lesion. Two‐thirds of MRI manifestations in asymptomatic PML are unilateral [15] and ICANS‐associated WM abnormalities are typically bilateral [16]. Our view is that unilateral MRI lesions in the setting of ICANS should be followed up with interval imaging to exclude other emerging pathologies.

PML‐IRIS is the paradoxical clinical deterioration observed in PML following immune reconstitution and is associated with specific neuroradiological features, particularly contrast enhancement on MRI [10]. Our patient developed radiological (but not clinical) PML‐IRIS after a single dose of pembrolizumab followed by clinical improvement, consistent with (i) other reports using checkpoint inhibition, where early radiological IRIS without clinical deterioration was observed [6, 8, 17] and (ii) studies in HIV‐positive patients demonstrating better prognosis post‐PML‐IRIS [10].

PD‐1 blockade is a promising novel therapeutic approach for PML [6, 7, 8, 9], but there is not yet consensus on its role [6, 18, 19, 20]. Our patient did not develop any clinical complications of pembrolizumab or recrudescence of CAR‐T toxicity and achieved a remarkable neurological recovery following 3 doses.

Whilst further studies are required, our case suggests that PD‐1 blockade may be a well‐tolerated and efficacious treatment for PML in the post‐CAR‐T setting.

## CONFLICT OF INTEREST

The authors declare that they have no conflict of interest.

TM, KT and CR are supported by the UK National Institute of Health Research University College London Hospital Biomedical Research Centre (BRC).

## AUTHOR CONTRIBUTIONS

Strachan Mackenzie wrote the manuscript. Manar Shafat performed T‐cell laboratory work. Harriet Roddy performed qPCR laboratory work. Harpreet Hyare reviewed MRI scans, produced the MRI image panel and edited the manuscript. Lorna Neill provided figure images and edited the manuscript. Michael Gilhooley reviewed and produced visual field analysis images. Eleanna Kara and Teresa Marafioti reviewed histopathology and provided figure images and legends. Emilie Sanchez, Maria A. V. Marzolini, Jeremy Rees, David S. Lynch, Kirsty Thomson, Kirit M. Ardeshna, Arian Laurence, Karl S. Peggs and Maeve O'Reilly reviewed and edited the manuscript. Claire Roddie was the lead author and edited the manuscript.

## Supporting information



Supporting information
