# Supplementary material for: Pembrolizumab for the treatment of progressive multifocal leukoencephalopathy following anti‐CD19 CAR‐T therapy: a case report
Source: EJHaem. 2021 Aug 4;2(4):848–53. doi: 10.1002/jha2.274 (PMC9281485; doi:10.1002/jha2.274)
Supplement: Supplementary file 1 — Supporting information [file JHA2-2-848-s001.docx]

**Supplementary data**

**Figure S1. Humphrey automated visual field plots.** OS = oculus sinister, left eye; OD oculus dexter, right eye. Panel A shows left-sided incongruous homonymous hemianopia at PML diagnosis, with near-complete resolution at 4.5 months after first infusion of Pembrolizumab (B). Cross hair gradations represent 10 degrees of visual field from the point of fixation; images attained on 24-2 SITA protocol on a Humphrey II. automated static perimeter (Zeiss, Oberkochen, DE).

**Figure S2. Constructional apraxia**. The inability to accurately copy drawings (commonly seen in parietal lobe syndromes) was detected on MMSE at initial presentation and was consistent with the parietal lobe involvement on MRI. Similar to other parietal syndromes, subtle apraxia persisted after initial problems such as visuospatial neglect had resolved. Whilst there was vast improvement by month 9 (above), note the persistent deficit (only 1 intersect of pentagons instead of 2), which resulted in an ongoing MMSE score of 29/30 (Figure 1B).

**Figure S3.** **Serial CAR-T qPCR levels.** qPCR levels from day of CAR-T infusion (Day 0) to 8 months post axi-cel infusion showing good early expansion and ongoing persistence (AUC 175,055 copies/μg DNA). There was no detectable increase in CAR-T levels following the pembrolizumab infusions (red arrows).
